# Efficient Ex Vivo Screening of Agents Targeting Thrombospondin1-Induced Vascular Dysfunction Using a Digital Multiwire Myograph System

**DOI:** 10.3390/mps4040074

**Published:** 2021-10-14

**Authors:** Molly Yao, Samayita Ganguly, Jane Hae Soo Shin, Tamer Elbayoumi

**Affiliations:** 1 Department of Pharmaceutical Sciences, College of Pharmacy-Glendale, Midwestern University, Cholla Hall 216, 19555 N. 59th Ave., Glendale, AZ 85308, USA; sgangu@midwestern.edu; 2 College of Graduate Studies, Midwestern University, Science Hall, 19555 N. 59th Ave., Glendale, AZ 85308, USA; 3 Arizona College of Osteopathic Medicine, Midwestern University, Glendale Hall, 19555 N. 59th Ave., Glendale, AZ 85308, USA; jane_hae_soo.shin@midwestern.edu

**Keywords:** myograph, vascular tone, isolated tissue, antagonist

## Abstract

Homeostasis of vascular tone is intricately and delicately maintained systemically and locally, by autonomic nerves and hormones in the blood and by intimal vasoactive substances, respectively. The balance can be acutely or chronically interrupted secondary to many alterations, especially under pathological conditions. Excessive matricellular glycoprotein thrombospondin 1 (TSP1) levels in circulation have been found to play an important role in ischemia-reperfusion injuries of different organs, by acutely suppressing vasorelaxation and chronically remodeling vascular bed. Our laboratory has been interested in identifying new drug moieties, which can selectively and effectively counteract TSP1-induced vascular dysfunction, in order to address associated clinical complications. Preliminary studies using computational docking and molecular models revealed potential drug candidates for further evaluation via vascular functional bioassay to prove the antagonism using an ex vivo vascular model. Herein, we described an efficient screening method for the identification of active drug candidates, by adapting a multiwire myograph system to perform a protocol with different treatments, in the presence of pathological levels of TSP1. We discussed the promising pharmacological evaluation results and suggested suitable modification for versatile applications. We also described the necessity of pre-determination of optimal resting tension to obtain the maximal response, if the experimental test model is different from those with determined optimal resting tension.

## 1. Introduction

Vascular tone, mediated by smooth muscle cells in the tunica media, is finely balanced among endothelium-derived vasoactive substances including vasodilators and vasoconstrictors. For example, endothelial nitric oxide synthase (eNOS) can be activated by shear stress or neurotransmitter acetylcholine to produce vasodilating nitric oxide (NO) [1,2]. Other vasoactive substances such as vasodilating prostacyclin and vasoconstrictive thromboxane A2, produced by cyclooxygenase, and 12-hydroxyeicosatetraenoic acids by lipoxygenase enzymes in the intimal layer, also contribute to homeostatic regulation of the local vasomotor tone. Endothelial dysfunction can be caused by aging, medical conditions, or agents that modulate activity or expression of eNOS and/or downstream effectors [3,4]. It is well known that an excess in matricellular protein thrombospondin 1 (TSP1) modulates the homeostasis of vascular tone by inhibiting eNOS activity and soluble guanylyl cyclase (sGC), as well as promoting the production of reactive oxygen species, which further reduce NO bioavailability [5,6,7]. Cumulative evidence demonstrated that pathological plasma levels of TSP1, via direct binding to its cognate cell membrane receptor CD47, play a pivotal role in pathogenesis and progress of a variety of clinical conditions such as ischemia-reperfusion injury and pulmonary arterial hypertension [8,9,10,11]. Other than CD47, TSP1 has also been found to interact with signal-regulatory protein α (SIRPα) to acutely inhibit vasorelaxation and chronically participate in tissue remodeling [12]. While accumulative mechanisms of TSP1 in underlying clinical conditions are revealed, the area of cardiovascular TSP1-targeting medicines remains to be tackled. Our laboratory has been interested in the identification of a new drug approach to selectively intervene with TSP1-mediated modulation of vascular tone [13].

Employing in silico simulations and molecular models, preliminary studies indicated several molecules can possibly be associated with the target protein TSP1 [14,15,16], which underwent subsequent functional bioassays to identify the potential drug candidate. Because TSP1 was proved to suppress endothelium-dependent vasorelaxation by intruding NO bioavailability and NO-initiated signaling cascade, the current study protocol design focused on endothelium-derived NO and consequential vascular tone. Cyclooxygenase and 5-lipoxygenase inhibitor meclofenamate sodium was included throughout the process to minimize fluctuation in the vasoreactivity caused by arachidonic acid cascade-produced vasoactive substances [17].

Commercially available myograph systems such as multiwire myograph and pressure myograph by Danish Myo Technology A/S (DMT, Hinnerup, Denmark), combined with a digital data acquisition device and a data recording and analysis software, enable to access the vasomotor function under different conditions and/or treatments with high sensitivity and efficiency. The protocol described herein used a two-pin multiwire automated myograph to evaluate responsiveness in isolated murine thoracic aorta, with a focus on the potential efficacy and potency of screened/selected candidate molecules as direct antagonists of/against TSP1-blunted endothelium-dependent vasorelaxation. C57BL/6J mouse, a common animal model used for the creation of transgenic or treatment-induced vascular disease murine in vivo models, was utilized in the present study. In spite of a distinct mechanism in regulating the vasoreactivity in the conduit vessel such as thoracic aorta from that in the resistance vessels like renal arterioles, others revealed that TSP1 signaling leads to resistance vessel dysfunction in the same manner as the systemic vasculature, altogether by debilitating vasodilation and enhancing vasoconstriction [7,12,18]. Therefore, isolated murine thoracic aorta was scientifically appropriate to be applied in the present study. Additionally, murine thoracic aorta held several technical advantages such as straightforward tissue harvesting, preparation, and abundance of functional tissues that can be successfully collected from a single experimental animal. This is in comparison with the typical scarcity of other usable narrower vascular tissues available from experimental animals, and the challenging skills to identify and harvest small resistance arterioles, such as mesenteric arterioles and pulmonary artery.

## 2. Experimental Design

### 2.1. Materials

- Native TSP1 protein, human (Novus Biologicals, Centennial, CO, USA; Cat No.: NBP1-99789; Thermo Scientific, Denver, IL, USA; Cat No.: RP-43151; Athens Research and Technology, Athens, GA, USA; Cat No.: 16-20-201319)
- CD47 antibody, clone mIAP301 (Novus Biologicals, Centennial, CO, USA; Cat No.: NBP1-43407)
- SIRPα antibody, clone C20 (Santa Cruz, Santa Cruz, TX, USA; Cat No.: sc-6922)
- Dextrose (Fisher Scientific, Tempe, AZ, USA; Cat No.: D-16-500)
- Calcium chloride dehydrate (Fisher Scientific, Tempe, AZ, USA; Cat No.: C614-500)
- Sodium bicarbonate (Fisher Scientific, Tempe, AZ, USA; Cat No.: S233-500)
- Potassium phosphate (Sigma-Aldrich, Saint Louis, MO, USA; Cat No.: P5379)
- Magnesium phosphate (Sigma-Aldrich, Saint Louis, MO, USA; Cat No.: M7506-500G)
- Potassium chloride KCl (EM Science, MA, USA; Cat No.: 7300)
- Sodium chloride (Sigma-Aldrich, Saint Louis, MO, USA; Cat No.: S5886)
- EDTA calcium Disodium salt (Santa Cruz, Santa Cruz, TX, USA; Cat. No.: SC-239970)
- Phenylephrine hydrochloride (Sigma-Aldrich, Saint Louis, MO, USA; Cat No.: P6126)
- Acetylcholine chloride (Sigma-Aldrich, Saint Louis, MO, USA; Cat No.: A6625)
- Forskolin (Sigma-Aldrich, Saint Louis, MO, USA; Cat No.: F6886)
- DMSO (Fisher Scientific, Tempe, AZ, USA; Cat No.: BP231-100)
- Meclofenamate sodium (Santa Cruz, Santa Cruz, TX, USA; Cat No.: SC-200532A)
- 0.9% saline (Fisher Scientific, Tempe, AZ, USA; Cat No.: Z1376)
- Isoflurane (Henry Schein, Melville, NY, USA; Cat No.: NDC11695-6776-1)
- 95% O_2_/5% CO_2_ (Airgas, Phoenix, AZ, USA; Cat No.: SGMOX5CO22)

### 2.2. Equipment

- Automated Multi Myograph System—620M (Danish Myo Technologies, DMT, Ann Arbor, MI, USA)
- PowerLab 8/35 (ADIntruments, Colorado Springs, CO, USA; Cat No.: PL3508)
- Force Calibration Kit (Danish Myo Technologies, Ann Arbor, MI, USA; Cat No.: 300041)
- Dissecting scope (Carl Zeiss Microscopy, White Plains, NY, USA; Cat No.: SteREO Discovery V8 with CL 1500 ECO)
- Dissecting dish (Living Systems Instrumentation, San Antonio, TX, USA; Cat No.: DD-90-S-BLK)
- Dumont #5 forceps (Fine Science Tools, Foster City, CA, USA; Cat No.: 11252-20)
- Dumont #7 forceps (Fine Science Tools, Foster City, CA, USA; Cat No.: 11271-30)
- Tissue forceps (Fine Science Tools, Foster City, CA, USA; Cat No.: 11021-12)
- Moria Miniature Vannas—curved (Fine Science Tools, Foster City, CA, USA; Cat No.: 15396-01)
- Vannas scissors—straight (Fine Science Tools, Foster City, CA, USA; Cat No.: 15009-08)
- Surgical scissors straight sharp/blunt (Fine Science Tools, Foster City, CA, USA; Cat No.: 14001-16)
- Surgical scissors straight sharp (Fine Science Tools, Foster City, CA, USA; Cat No.: 14002-13)
- Surgical tape (Various suppliers)
- 30G × 1 in needle (BD, NJ, USA; Cat No.: 305128)
- 3 mL syringe (BD, NJ, USA; Cat No.: 309657)
- Water bath with immersion circulator (Cole-Parmer, Vernon Hills, IL, USA; Cat No.: EW-12120-13)
- Alternate option to water bath: automatic buffer filler system—625FS (Danish Myo Technologies, Ann Arbor, MI, USA)
- Microfuge tube (Fisher Scientific, Phoenix, AZ, USA; Cat No.: 02-682-550)

### 2.3. Software

- LabChart 8 (ADInstruments Version 8)
- GraphPad Prism version 8.0 (GraphPad Software Inc., San Diego, CA, USA)

## 3. Procedure

### 3.1. Preparation of the Instrument

- Optional: Myography Calibration

Weight calibration is recommended at least once a month for wire myography by the manufacturer. A step-by-step procedure is included in the FORCE CALIBRATION sub-menu under SETTING and detailed in the manual [19]. Following calibration of each channel, remember to conduct a unit conversion in the software LabChart 8, which allows to convert the unit of tension in Newton showed in myography to milligram. A tutorial video demonstrating the complete process of myograph calibration and subsequent unit change for each channel can be found on the ADInstruments website [20]. Save the file as Chart Setting File (*.adiset) and mark the date of calibration.

- On the day of experiment, power on PowerLab and myograph with HEAT OFF, and leave them on standby. Open the most recent calibration file in LabChart.

### 3.2. Preparation of Buffers

#### 3.2.1. Preparation of Stock Solution

- **KCl Stock**

Dissolve 22.365 g of KCl in 100 mL of ddH_2_O, to make a stock solution with a concentration of 223.65 g/L or 3 M, and store at room temperature.

- **Foskolin Stock**

Dissolve 5 mg of forskolin in 1 mL of DMSO, to make a stock solution with a concentration of 5 mg/mL or 12.18 mM, which is stable at room temperature for at least 6 months.

Optional stock solutions for fresh Krebs–Henseleit solution preparation.

- **Krebs Stock Salts**

Fill a 2 L Erlenmeyer flask or beaker with 1 L of ddH_2_O, dissolve 101.3 g of NaCl, 4.67 g of KCl, 1.88 g of MgSO_4_, and 2.37 g of CaCl_2_. Transfer to a storage container and store at 4 °C.

Alternately, 3.845 g of MgSO_4_·7H_2_O or 3.137 g of CaCl_2_·2H_2_O can be used.

- **Krebs Stock Phosphate**

Dissolve 6.69 g of KH_2_PO_4_ (monobasic) in 500 mL of ddH_2_O, to make a stock solution with a concentration of 13.38 g/L or 1.18 mM, and store at 4 °C.

- **Krebs Stock EDTA**

Dissolve 0.973 g of Na_2_Ca EDTA·nH2O in 100 mL of ddH_2_O, to make a stock solution with a concentration of 9.73 g/L or 26 mM, and store at 4 °C.

#### 3.2.2. Preparation of Fresh Solution

- **Fresh Meclofenamate working stock**

On the day of the experiment, dissolve 31.81 mg of meclofenamate in 10 mL of ddH_2_O/ethanol (50%/50% *v*/*v*), to make a stock solution with a concentration of 1 × 10^−2^ M, and keep at room temperature until use. Leftover is disposed.

- **Fresh phenylephrine hydrochloride (PE) and acetylcholine chloride (Ach) working stocks**

1. Dissolve 20.37 mg of PE or 18.17 mg of Ach into 1 mL of ddH_2_O to make 1 × 10^−2^ M PE or 1 × 10^−2^ M Ach stock solutions, respectively.
2. Make 10× serial dilution of PE and Ach working stocks by mixing 20 µL of PE or Ach and 180 µL of Krebs buffer. For example, 20 µL of 1 × 10^−1^ M PE is mixed with 180 µL of Krebs buffer to make 200 µL of 1 × 10^−2^ M PE.

#### 3.2.3. Fresh Krebs–Henseleit Solution

Fresh Krebs buffer can be prepared with Krebs stock salts, Krebs stock phosphate, and Krebs stock EDTA.

1. To prepare 1 L of fresh Krebs buffer, fill a calibrated Erlenmeyer flask with 750 mL of ddH_2_O, dissolve 1.25 g of NaHCO_3_ and 0.99 g of dextrose, followed by 12 mL of KH_2_PO_4_ stock and 1 mL of EDTA stock, while stirring.
2. Gas the solution with 95% O_2_/5% CO_2_, gradually add 75 mL of stock salts.
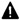
**CRITICAL STEP** Slow addition of stock salts while stirring and bubbling is required to prevent precipitation.
3. Add the remaining volume of 162 mL of ddH_2_O to make a final volume of 1 L, and add 310 µL of meclofenamate stock. Continue bubbling for around 5 min, seal the flask with parafilm, and store at 4 °C until use.

**OPTIONAL STEP** Fresh Krebs–Henseleit Solution can be alternatively prepared as follows, without using stock solutions mentioned in step 1 and 2. Fill a calibrated Erlenmeyer flask with 1 L of ddH_2_O, dissolve 1.25 g of NaHCO_3_ and 0.99 g of dextrose, 7.6 g of NaCl, 350.4 mg of KCl, 140.8 mg of MgSO_4_ (or 288.4 mg of MgSO_4_·7H2O), 177.6 mg of CaCl_2_ (or 235.2 mg of CaCl_2_·2H2O), 160.6 mg of KH_2_PO_4_ (monobasic), and 9.7 mg of Na_2_Ca EDTA·nH_2_O. Add 310 µL of freshly prepared meclofenamate stock, seal the flask with parafilm, and store at 4 °C until use.

Both preparations concordantly produce a standard Krebs buffer (composition in mM: NaCl, 118.4; KCl, 4.7; CaCl_2_, 1.6; MgSO_4_, 1.2; KH_2_PO_4_, 1.2; NaHCO_3_, 14.9; dextrose, 5.5; Na_2_Ca EDTA, 0.026; meclofenamic acid, 0.0031; pH 7.4).

#### 3.2.4. TSP1 Stock (Novus or Athens) Preparation

1. Spin down.
2. Add 270.6 µL of ddH_2_O to the vial containing 25 µg of TSP1, vortex to completely dissolve, and spin down. An aliquot of 50 µL should be kept and used on ice, with the rest stored at −20 °C.

#### 3.2.5. Drug Candidate Preparation

Dissolve drug candidate in ddH_2_O to make a stock solution, make an aliquot of 15 µL, and store at −20 °C. Dilute the stock solution to the concentrations to be tested and keep on ice. For example, a working stock of 6.6 × 10^−6^ M was prepared to reach a final concentration of 6.6 × 10^−9^ M in this protocol. Discard the leftover.

### 3.3. Tissue Preparation and Mounting of the Artery

**Figure 1:**
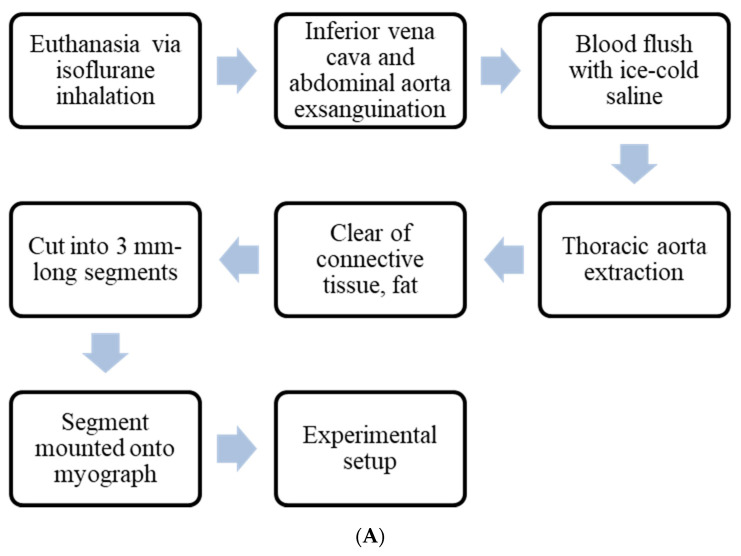

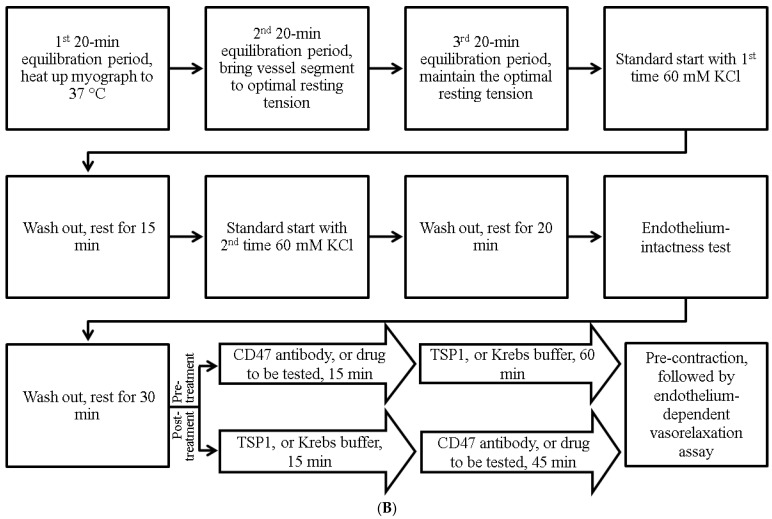
A complete process flow diagram of the experimental setup. (**A**) Workflow starting from tissue harvest until mounting of isolated vascular tissues onto myograph. (**B**) Experimental setup procedure for screening of proposed drug candidates using an ex vivo model, along with appropriate controls.

The procedure starting from animal euthanasia until tissue mounting is explicitly demonstrated in Figure 1A.

#### 3.3.1. Animal Dissection

1. Euthanasia with isoflurane inhalation.
2. Lay the mouse on its back onto a blue under pad. Tape down paws for a complete exposure of the ventral side.
3. Wipe the chest and abdomen with Kim wipes wet with water or alcohol.
4. Grasp the skin with tissue forceps, cut open the abdomen with straight sharp/blunt surgical scissors, and exsanguinate by cutting off the abdominal aorta and inferior vena cava with straight sharp surgical scissors.
5. Cut open the rib cage with straight sharp/blunt surgical scissors to reveal the chest cavity. Slowly inject 3 mL of cold saline into the left ventricle through the heart apex. The lung turns from pink to whitish with blood flush-out.

#### 3.3.2. Tissue Harvest

1. Add freshly prepared cold Krebs buffer into a dissecting dish. Generally, 3–5 mL is enough for immersion of a harvested vessel. Adjust the volume according to the size of your dissecting dish. Set aside, preferably on ice.
2. Add 5 mL of freshly prepared cold Krebs buffer into myograph chamber and gas with 95% O_2_/5% CO_2_. Keep the myograph remaining in HEAT OFF.
3. Remove the lungs and heart to expose the descending thoracic aorta.
4. From the right or the left side of the body, slide the curvy part of a Dumont #7 forceps underneath the aorta and make sure to lay the forceps against the spine.
5. Repeat closing and releasing the forceps to loosen the connective tissue/fat between the aorta and spine. Repeat step 4 and 5 along the thoracic aorta.
6. Make a cut at the aorta hiatus at the diagram with straight Vannas scissors.
7. Grab the fat tissue on top of the loose end of the aorta with a Dumont #5 forceps in the left hand, and slightly raise the loosened tissue. Start cutting with a curved Moria Miniature Vannas in the right hand from the loose end up to the neck.

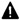
**CRITICAL STEP** With more aortic tissue cut off from the spine, gently raise the loose aorta for better vision as well as prevention of accidental poke into the vessel wall. Do not pull or stretch the loose aorta to avoid potential injury to the endothelium. Moreover, make sure the scissor tips are moving against the spine to reduce the risk of cutting into the vessel wall.

#### 3.3.3. Tissue Preparation and Mounting

1. Immerse the isolated aorta into cold Krebs solution in the dissecting dish. Secure the aorta by pinning down fat/connective tissues at two ends of the isolated vessel. Fasten an approximately 2 cm long plastic ruler along the isolated vessel.
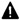
**CRITICAL STEP** Do not stretch the isolated vessel to avoid injury or damage to the intimal layer of the vessel, especially when the vasoreactivity of the endothelium is of interest.
2. Clear off fat/connective tissues by gently pulling the fat/connective tissues away from the vessel using Dumont #5 forceps and cutting it off with a curved Moria Miniature Vannas whose tip is kept away from the vessel. The vessel branching remains uncut for the purpose of seizing in step 5. The fat/connective tissue at two ends generally is not used owing to possible injury during the process of harvest.
3. Cut the aorta into segments 2.5 to 3 mm in length with straight Vannas scissors, starting at least 1 mm from each end. Keep aortic segments in cold Krebs buffer until use.
4. Remove a chamber off the myograph interface. Adjust the micropositioner of the myograph chamber to bring the two pins as close together as possible, but not touching each other, under the dissecting microscope.
5. Hold a vessel branching of one aortic segment with Dumont #5 forceps to transfer it from the dissecting dish to the myograph chamber and mount it onto pins.
6. Adjust the micropositioner to slightly stretch the mounted vascular segment in order to prevent it from sliding off. Install the chamber back onto the myograph interface, resume gas, and continue keeping HEAT OFF. A vessel segment mounted onto myograph and stretched to the optimal resting tension is exhibited in step 2 of Section 3.4.1 Establishment of optimal resting tension.
7. Repeat steps 4 to 6 for the remaining vessels.

### 3.4. Experiment

The process of the experimental setup is displayed in Figure 1B.

#### 3.4.1. Establishment of Optimal Resting Tension

**Figure 2:**
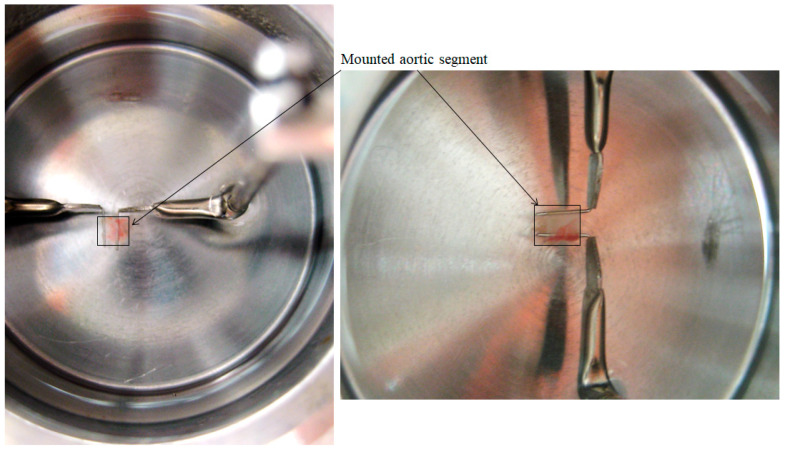
Isolated murine thoracic aorta segment, gradually stretched to the optimal resting tension of 500 mg.

1. After all vessel segments are mounted, click “start” in LabChart to start recording; zero all channels following the direction shown on myograph display screen. Turn on myograph heat, and proceed to experiment setup by beginning timing. Turn on the water bath to warm up Krebs buffer with continuous gassing with 95% O_2_/5% CO_2_, if DMT Automatic Buffer Filler System—625FS is not available. The vessels segments are incubated in warming up Krebs buffer for 20 min, counted as the first 20 min equilibration period.
2. Change buffer. Start timing for the second 20 min equilibration period. Zero all channels. Gradually stretch vessel rings by adjust micropositioner to add resting tension to the vessels. Resting tension starts from 0 to 500 mg, with a 100 mg increase and an interval of 30 s between each stretch, while closely monitoring reading changes in the LabChart interface. Continue adjusting every few minutes until the tension stays constant at 500 mg (Figure 2).

3. Change buffer to start the third 20 min equilibration period. Adjust tension to 500 mg if necessary. Until now, the aortic segments are incubated for a total of 60 min.

#### 3.4.2. Standard Start (Also Called Tissue Priming)

1. Add exactly 5 mL of Krebs buffer to each myograph chamber and adjust the micropositioner to maintain the resting tension at 500 mg. Zero all channels.
2. Add 100 µL of 3 M KCl stock to make a final concentration of 60 mM in each chamber, and mark in LabChart. Wait for 5–10 min and wash out three times with approximately 5 mL of Krebs buffer. If the tension falls below 0, bring it back up to 0 mg, and allow the vessel rings to relax for 10–15 min.
3. Repeat the second KCl stimulation for 20 min, followed by washout three times and relaxation for 20 min. Adjust tension to 0 mg, if necessary.

#### 3.4.3. Endothelium Intactness Examination

1. Add exactly 5 mL of Krebs buffer to each chamber and adjust the resting tension to 0 mg, if necessary. Add 5 µL of 1 × 10^−3^ M PE to each chamber and mark in LabChart. Wait for the PE-induced contraction to reach a plateau, and write down the force of tension.
2. Without changing the buffer, add 5 µL of 1 × 10^−3^ M Ach to each chamber and mark in LabChart. Wait for Ach-induced relaxation to reach a plateau, and write down the force of tension. Calculate the percentage of relaxation induced by Ach with the following equation:

$$\%\;of\;relaxation=\frac{force\;in\;step\;1-force\;in\;step\;2}{force\;in\;step\;1}\times 100\%$$

Only the vessel rings demonstrating 80% of relaxation or greater are selected for further experiment.

3. Proceed to washout three times. Let the vessel rings relax for 30 min with replacement of Krebs buffer every 10 min. Adjust the resting tension to zero if necessary.
4. Proceed to the next experiment setup, described under either Section 3.4.4.1 Pharmacological screening of potential drug candidates/pre-treatment experiment, or Section 3.4.4.2 Therapeutic treatment experiment/post-treatment experiment, as needed.

#### 3.4.4. Experiment

##### 3.4.4.1. Pharmacological Screening of Potential Drug Candidates/Pre-Treatment Experiment

1. Add exactly 5 mL of buffer to each chamber.
  Note: The volume of Krebs buffer may need to be adjusted according to the volume of pharmacological agent to be added, in order to ensure the concentration accuracy of the pharmacological agent. Our laboratory applies a volume rule of 1% or less. For instance, the volume of Krebs buffer is reduced to 4950 µL when 50 µL of TSP1 is added to a myograph chamber.
2. Add 10 µL of CD47 antibody, 10 µL of SIRPα antibody, or 5 µL of selected drug candidate to make a final concentration of 1 µg/mL, 2 µg/mL, or 6.6 × 10^−9^ M, noted as dose 1, respectively. Mark the treatment at the corresponding channel in LabChart. Start the timer.
  Note: This step was skipped in the tests of reactivity of TSP1 from different vendors.
3. Precisely 15 min later, add 50 µL of TSP1 into three chambers pre-treated with CD47 antibody, SIRPα antibody, or the drug candidate, or Krebs buffer only, respectively. TSP1 incubation time is set at 60 min. Generally, a fourth vessel segment is left untreated as a negative control.
  Note: Volume of TSP1 needs to be adjusted based on the concentration of original package or stock solution.
4. Mark treatment at corresponding channels in LabChart.
5. Add 5 µL of 1 × 10^−3^ M PE into each chamber without changing the buffer, mark in LabChart, and wait for PE-induced contraction to reach a plateau.
6. Start Ach doses, starting from a final concentration of 1 × 10^−9^ M up to 1 × 10^−5^ M, cumulatively in half-log increments, following the Table 1. Mark each dose of Ach in LabChart.
  **OPTIONAL STEP** At the end of the experiment, add 4.1 µL of 12.18 mM forskolin to the vessel ring received TSP1 treatment only to make a final concentration of 1 × 10^−5^ M. Forskolin is included occasionally to prove the viability of the vessel, which does not respond to Ach stimulation.
7. Click “Stop” in LabChart. Save the file for further analysis. Generally, the file name includes experiment date, treatment, and dose. If the name is too long, make a note in the lab notebook with details.
8. Proceed to Section 3.4.5 Clean up.

##### 3.4.4.2. Therapeutic Treatment Experiment/Post-Treatment Experiment

1. Add exactly 5 mL of buffer to each chamber.
  Note: The rule of thumb of 1% or less in volume consistently applies. The volume of Krebs buffer may need to be adjusted according to the volume of pharmacological agent to be added, in order to ensure the concentration accuracy of the pharmacological agent.
2. Add 50 µL of TSP1 into three chambers to be treated with CD47 antibody, or the drug candidate, or Krebs buffer only, respectively. Mark the treatment at the corresponding channel in LabChart. Start the timer. TSP1 incubation time is set for 60 min. Generally, a fourth vessel segment is left untreated as a negative control.
  Note: Volume of TSP1 needs to be adjusted based on the concentration of original package or stock solution.
3. Precisely 15 min later, add 10 µL of CD47 antibody, 10 µL of SIRPα antibody, or 10 µL of selected drug candidate to make a final concentration of 1 µg/mL, 2 µg/mL, or 1.32 × 10^−8^ M, noted as dose 2, respectively.
  Note: This step was skipped in the tests of reactivity of TSP1 from different vendors.
4. Mark each dose treatment at the corresponding channels in LabChart. The co-incubation period for CD47 antibody, SIRPα antibody, or drug candidate with TSP1 is 45 min.
5. Add 5 µL of 1 × 10^−3^ M PE into each chamber without changing the buffer, mark in LabChart, and wait for PE-induced contraction to reach a plateau.
6. Start Ach doses, starting from a final concentration of 1 × 10^−9^ M up to 1 × 10^−5^ M, cumulatively in half-log increments, following Table 1. Mark each dose of Ach in LabChart.
  **OPTIONAL STEP** At the end of the experiment, add 4.1 µL of 12.18 mM forskolin to the vessel ring receiving TSP1 treatment only to make a final concentration of 1 × 10^−5^ M. Forskolin is included occasionally to prove the viability of the vessel, which does not respond to Ach stimulation.
7. Click “Stop” in LabChart. Save the file for further analysis. Generally, the file name includes experiment date, treatment, and dose. If the name is too long, make a note in the lab notebook with details.
8. Proceed to Section 3.4.5 Clean up.

#### 3.4.5. Clean Up

Remove the vessel rings and dispose properly. Flush and clean the myograph systems according to the manufacturer’s instructions.

### 3.5. Data Analysis

1. Open data file in LabChart and quantify vessel contractile/relaxing responses using the Dose Response Module. A step-by-step video instruction on how to use the Dose Response Module in LabChart is available [21], which reports a variety of pharmacological parameters such as half maximal effective concentration (EC50) and hill slope.
2. Create a new GraphPad file and copy the dose and matching force to the GraphPad file for statistical analysis. Concentration–response curves were plotted using non-linear regression fitting. Comparisons of concentration–response curves between the groups were performed using two-way analysis of variance (ANOVA) followed by a Bonferroni post hoc test. The logarithms of EC50 values were compared using a two-tailed Student’s *t*-test, and a *p*-value smaller than 0.05 was taken as significant.

## 4. Representative Results

### 4.1. Examination of Bioactivity of TSP1

Native TSP1 is widely expressed in human tissues with low tissue specificity and is marginally detected in normal subjects. However, upregulation of tissue TSP1 and/or circulating TSP1 can be found to be associated with various organ injuries and chronic clinical conditions [22,23,24,25,26]. TSP1 was first known to be stored and released from platelet α-granules upon activation [27,28]. Most of the commercially available TSP1 protein is extracted from human platelets. Depending on the methods applied for its extraction and purification as well as the quality of storage conditions, the bioactivity of extracted native TSP1 may vary considerably between manufacturing facilities and suppliers. In addition, commonly applied quality control criteria in the electrophoretic analysis of purified TSP1 product cannot verify most of the pharmacological activities of interest. Therefore, it was essential to examine the bioactivity of native human TSP1 available from different commercial vendors (Thermo Scientific, Novus Biologicals, and Athens Research & Technology), specifically in terms of modulation of vasoactivity, in order to determine which TSP1 protein products can be used for the candidate drug screening assays afterwards. To note, TSP1 proteins purchased from Thermo Scientific and Novus Biologicals (Pierce^TM^-TSP1 and Novus-TSP1, respectively) were purified from human platelets, while TSP1 protein obtained from Athens Research & Technology (Athens-TSP1) was purified from human blood/plasma.

**Figure 3:**
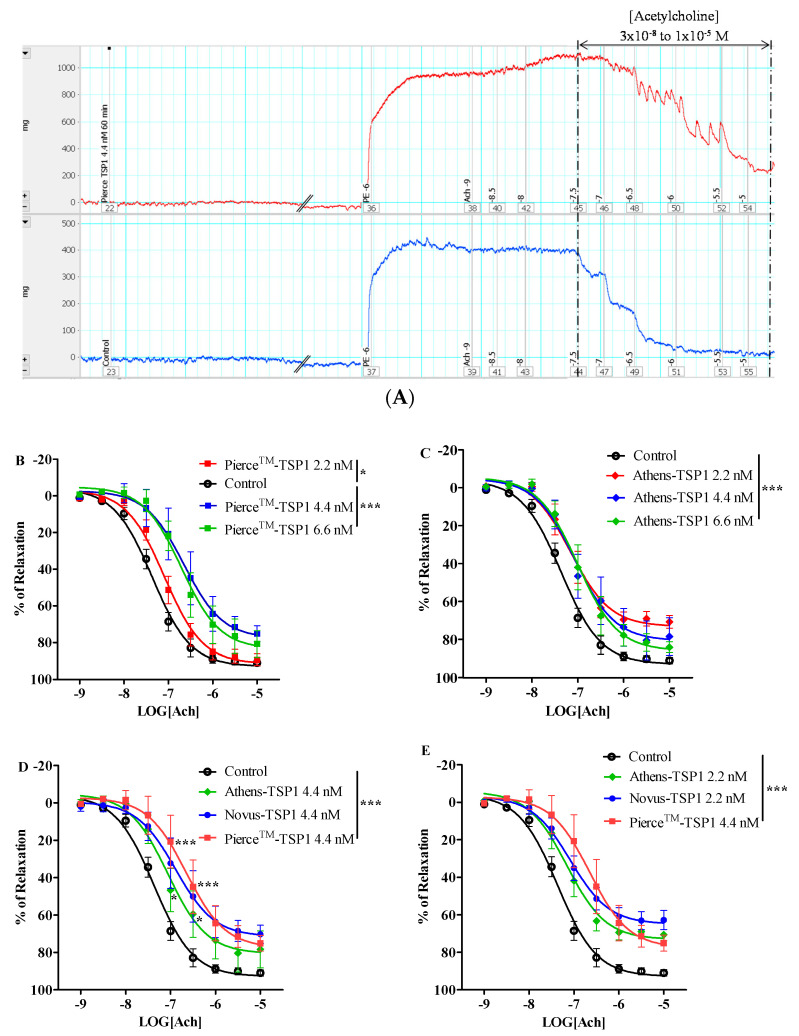
Native thrombospondin 1 (TSP1) from different sources inhibited endotheliumdependent vasorelaxation with different efficacies. (**A**) Representative force vs. time traces recordings of isolated murine thoracic aorta responses to phenylephrine followed acetylcholine stimulations in the presence of Pierce^TM^—TSP1 or vehicle control, i.e., Krebs buffer (PE: phenylephrine; Ach: acetylcholine; the numbers marked in the recording are the logarithm value of concentrations of the stimulants). Concentration response curves of isolated mouse thoracic aorta exposed to Pierce^TM^—TSP1 (**B**) or Athens—TSP1 (**C**) at 2.2, 4.4, or 6.6 nM. Pierce^TM^—TSP1 induced impairment of vasodilation in a slightly different pattern than Athens—TSP1 (*, *p* < 0.05, TSP1 vs. control; ***, *p* < 0.0001, TSP1 vs. control). (**D**) The same concentration TSP1, e.g., 4.4 nM, from three sources inhibited vasorelaxation, two of which produced greater suppression of vasorelaxation induced by acetylcholine 1 × 10^−7^ and 3 × 10^−7^ M (*, *p* < 0.05, Athens—TSP1 vs. control; ***, *p* < 0.0001, Pierce^TM^—TSP1 or Novus—TSP1 vs. control). (**E**) An equivalent inhibition of vasorelaxation was reached when Novus—TSP1 or Athens—TSP1 at 2.2 nM and Pierce^TM^—TSP1 at 4.4 nM was applied (***, *p* < 0.0001, TSP1 vs. control).

As evident from tension traces demonstrated in Figure 3A, 4.4 nM of Pierce^TM^—TSP1 enhanced vasoconstriction by inhibiting acetylcholine-induced endothelium-dependent vasorelaxation over a wide concentration range, starting at 3 × 10^−8^ M until 1 × 10^−5^ M. The concentration–response curve of vasorelaxation normalized to 1 × 10^−6^ M phenylephrine-induced vasocontraction in the presence of three different concentrations of Pierce^TM^—TSP1, specifically, 2.2, 4.4, and 6.6 nM, was first compared to determine if TSP1-inhibited vasorelaxation was indeed dose-dependent (Figure 3B). Pierce^TM^—TSP1, originating from blood/plasma, demonstrated a greater inhibition of vasorelaxation at 4.4 nM compared with that at 2.2 nM, while no significant difference in impaired vasorelaxation was observed at the highest Peirce^TM^—TSP1 at 6.6 nM, indicating reaching a plateau level in modulating vasoactivity. On the other hand, all three tested concentrations of Athens—TSP1, extracted from human platelet, showed almost the same level of inhibition of vasorelaxation (Figure 3C) (*p* ≥ 0.4). Likewise, the same pattern of concentration effect of TSP1 was also observed in Novus—TSP1 prepared from human platelet (data not shown). Furthermore, a comparison of the dose–response curves in the presence of the same concentration, e.g., 4.4 nM of TSP1, based on the three different TSP1 vendors, showed that Pierce^TM^—and Novus—TSP1 significantly impaired vasorelaxation up to almost the same extent. Both Pierce^TM^—TSP1 and Novus—TSP1 products had a significantly greater inhibition of vasorelaxation induced by 1 × 10^−7^ and 3 × 10^−7^ M acetylcholine, respectively, in comparison with Athens—TSP1 (Figure 3D). Comparison of all dose–response curves treated with different concentration of TSP1 from each individual vendor revealed that both Athens—TSP1 and Novus—TSP1 at 2.2 nM inhibited vasorelaxation almost equivalently to Pierce^TM^—TSP1 at 4.4 nM (Figure 3E). Equivalent and consistent dysregulation of vascular tone by pathological level of TSP1 from different vendors, serving as the negative control, is critical for the subsequent pharmacological screening process of anti-TSP1 drug candidates. Hence, the concentration of TSP1, per each individual vendor, obtained in this pre-screening step was selected to be repeatedly applied in the following pharmacological evaluation assay to minimize variation in the negative controls; for example, Pierce^TM^—TSP1 at 4.4 nM was applied hereafter.

### 4.2. Pharmacological Screening of TSP1-Targeting Drug Candidates

**Figure 4:**
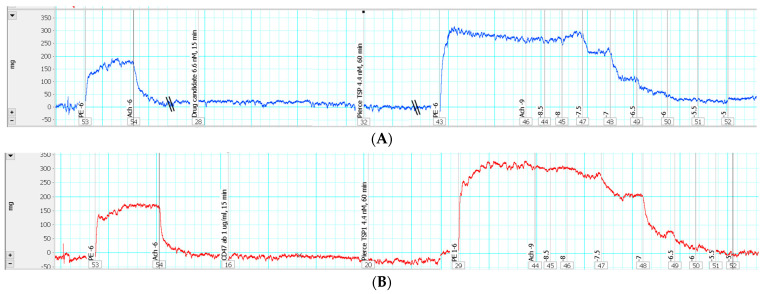

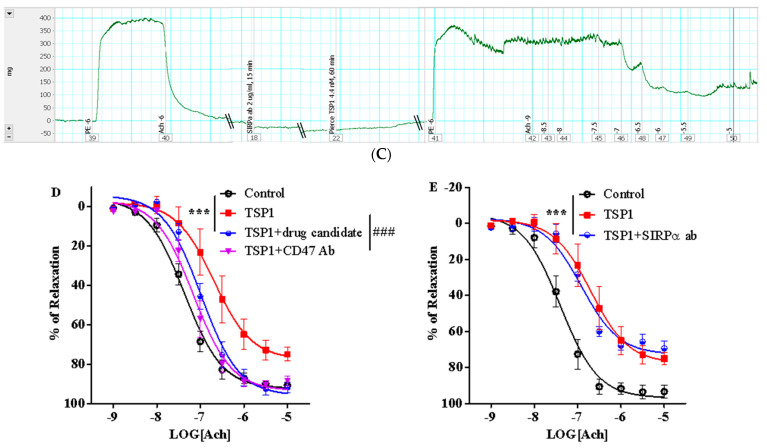
Thrombospondin 1 (TSP1)−impaired endothelium-dependent vasorelaxation was prevented by treatment with the proposed drug candidate, but not by blocking signal-regulatory protein α (SIRP α). Representative force vs. time traces recording from endothelium intactness test to acetylcholine-induced vasorelaxation by isolated murine thoracic aorta pre-treated with the proposed drug candidate 6.6 nM (**A**), CD47 antibody (CD47 ab) 1 µg/mL (**B**), or SIRPα antibody (SIRPα ab) 2 µg/mL (**C**) in the presence of Pierce^TM^—TSP1 4.4 nM (PE: phenylephrine; Ach: acetylcholine; the numbers marked in the recording are the logarithmic values of stimulant concentrations). Prior to receiving any treatment, the vessel segments were pre-contracted with 1 × 10^−6^ M PE followed by 1 × 10^−6^ M Ach-stimulated endothelium-dependent vasorelaxation to confirm endothelial intactness. (**D**) Concentration response curves of isolated mouse thoracic aorta exposed to TSP1, in the presence of vehicle, CD47-based drug candidate, or CD47 antibody. (**E**) Concentration response curves of isolated mouse thoracic aorta exposed to TSP1, in presence of vehicle or SIRPα antibody (***, *p* < 0.0001, TSP1, TSP1 + drug candidate, or TSP1 + SIRPα ab vs. control; ###, *p* < 0.0001, TSP1 + drug candidate or TSP1 + CD47 antibody vs. TSP1).

TSP1 is well known to induce inhibition of vasorelaxation via interaction with multiple cellular receptors/ligands. This protocol first described a pharmacological screening of two potential drug targets reported previously, i.e., CD47 and SIRPα. To determine the effectiveness of our newly identified CD47-based non-antibody drug candidate and SIRPα blocking antibody in antagonizing TSP1-induced suppression of vasorelaxation, a prophylactic treatment was applied to isolated murine thoracic aorta with intact endothelium, in the presence of a pathological level of TSP1 challenge (utilizing 4.4 nM of Pierce^TM^—TSP1). An evaluation of the two anti-TSP1 drug candidates was conducted against vehicle control and positive control with anti-mouse monoclonal CD47 antibody as well. Tension trace demonstrated that the vessel rings treated with a proposed CD47-based drug candidate (Figure 4A) were effectively protected from TSP1-mediated suppression of vasorelaxant response to acetylcholine, virtually equivalent to the protection produced by CD47 antibody (Figure 4B). In fact, treatment with CD47 blocking antibody or proposed CD47-derived drug candidate significantly counteracted TSP1-inhibited vasorelaxation (Figure 4D), completely or very close back to the vehicle control level, respectively. The maximum relaxation in response to the highest concentration of acetylcholine was restored from 75.1 ± 3.5%, in the presence of TSP1 only, up to 88.6 ± 2.5% and 92.0 ± 2.5%, following CD47 antibody or drug candidate treatment, respectively, i.e., reaching effectively the same vasorelaxation level of the vehicle control at 92.0 ± 1.4%. This prophylactic effect against TSP1-enhanced vasoconstriction was also supported by restoration of sensitivity to acetylcholine, comparable to the isolated vascular bed treated with CD47 blocking antibody. Specifically, TSP1 suppressed the vasodilation potency of acetylcholine from 43.5 ± 6.3 nM to 371.4 ± 153.9 nM, while pre-treatment with CD47 antibody or the CD47-based drug candidate successfully restored the native acetylcholine potency all the way back to 83.6 ± 17.6 nM or 117.8 ± 27.1 nM, respectively. The discussed bioassay protocol successfully determined the pharmacological efficacy of our proposed CD47-derived drug prototype to neutralize impaired vasorelaxation secondary to excess TSP1.

In contrast to the positive results obtained with the CD47-based drug candidate, SIRPα blocking antibody did not demonstrate any protective effect against TSP1-reduced vasorelaxation (Figure 4C). Decreased responses and sensitivity to acetylcholine in the presence of a pathological level of TSP1 remained the same for the vessels receiving SIRPα antibody treatment (Figure 4E). At this stage, pursuing specific SIRPα antibody and/or SIRPα-mediated interference as a pharmacological approach to target TSP1-imparied endothelium-dependent vasorelaxation does not seem sufficiently warranted.

### 4.3. CD47-Based Candidate Drug as a Potential Therapy

**Figure 5:**
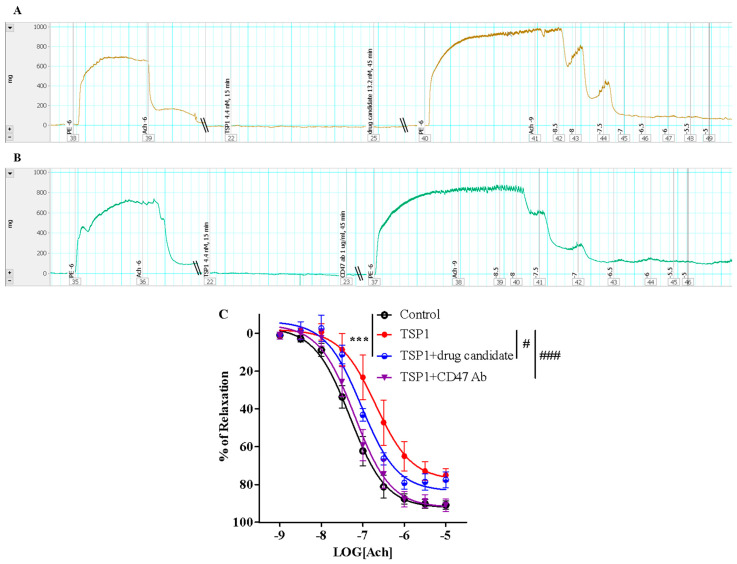
Acute TSP1 challenge-induced reduction in endothelium-dependent vasorelaxation was significantly restored by therapeutic treatment with the proposed CD47-based drug candidate. Representative force vs. time traces recording from endothelium intactness test to acetylcholine-induced vasorelaxation by isolated murine thoracic aorta exposed to acute TSP1 (Pierce^TM^—TSP1 4.4 nM) challenge for 15 min, followed by treatment with the proposed CD47-based drug candidate 13.2 nM (**A**) or CD47 antibody (CD47 ab) 1 µg/mL (**B**) for 45 min (PE: phenylephrine; Ach: acetylcholine; the numbers marked in the recording are the logarithmic values of stimulant concentrations). Prior to receiving any treatment, the vessel segments were pre-contracted with 1 × 10^−6^ M PE followed by 1 × 10^−6^ M Ach-stimulated endothelium-dependent vasorelaxation to confirm endothelial intactness. (**C**) Concentration response curves of isolated mouse thoracic aorta exposed to TSP1, in the presence of vehicle, CD47-based drug candidate, or CD47 antibody (***, *p* < 0.0001, TSP1 or TSP1 + drug candidate vs. control; #, *p* < 0.05, TSP1 + drug candidate vs. TSP1; ###, *p* < 0.0001, TSP1 + CD47 antibody vs. TSP1).

The successful prophylactic treatment of TSP1-reduced vasorelaxation encouraged further investigation into the CD47-based drug candidate as a therapy. Hence, original ex vivo protocols were modified into a pre-clinical set-up that mimics reduced vasorelaxation owing to a sudden pathological surge of TSP1, as demonstrated in disease conditions such as ischemia-reperfusion injury. An acute TSP1 challenge for 15 min, followed by therapeutic treatment with the drug candidate or CD47 antibody, was adopted by switching the sequence of administration of TSP1 and the drug candidate or CD47 antibody in the original pre-treatment protocol. Under this new post-treatment/therapeutic protocol setup, the vessels treated with the drug candidate showed restoration of responses to acetylcholine (Figure 5A) close to those treated with CD47 antibody (Figure 5B). Therapeutic post-TSP1 treatment with the CD47-based drug candidate restored vasodilation potency of acetylcholine back to 92.46 ± 20.8 nM, comparable to the potency of 63.95 ± 14.7 nM restored by CD47 antibody. However, at this tested dose, the drug candidate resulted in maximal vasorelaxation of 77.4 ± 9.8%, significantly less than that achieved through post-treatment with CD47 antibody at 90.91 ± 2.6% (Figure 5C). Increasing the post-treatment dose of the CD47-based drug candidate by just twofold demonstrated efficient recovery of maximal vasorelaxation equivalent to that achieved even at the control level (data not shown), thus indicating its potential to be a viable therapeutic approach to selectively target TSP1-modulated vascular tone [12].

## 5. Conclusions

With growing translational and clinical knowledge about TSP1 tissue levels in health and disease, its excess has been repeatedly shown to play pivotal pathological roles in multiple medical conditions. For example, TSP1 was found to participate in ischemia-reperfusion injury, both acutely, via impairment of vasorelaxation, and chronically, via remodeling of vascular bed, ultimately leading to irreversible damage to the ischemic and surrounding tissue or organ. Our laboratory has been interested in identifying a drug candidate to serve as an antagonist to counteract detrimental effects exerted by TSP1. To examine whether a pharmacological agent was effective in antagonizing TSP1-induced suppression of vasorelaxation, an ex vivo model of isolated thoracic aorta was utilized for an efficient vasoreactivity pharmacological assay, in order to practically mimic the acute ischemic scenario induced by presence of pathological levels of TSP1. Herein, two potential drug candidate strategies, based on knowledge of innate TSP1–CD47 receptor and TSP1–SIRPα protein interactions, were pharmacologically evaluated using this protocol.

The initial screening assay was conducted to determine the modulating effect on vasorelaxation by several commercial TSP1 protein products. TSP1 from three vendors replicated a significant inhibition of endothelium-dependent vasorelaxation, as published by others [5,10]. A maximal inhibitory effect by TSP1 was reached at 2.2 nM for Novus—TSP1 and Athens—TSP1 and 4.4 nM for Pierce^TM^—TSP1, respectively, in the preliminary screening process. Among the three tested TSP1 products, which demonstrated equivalent modulation of vascular tone, Pierce^TM^—TSP1 was selected owing to superior consistency in vasoactivity throughout all product batches. Thus, a fixed dose of 4.4 nM of Pierce^TM^—TSP1 was consistently applied into all subsequent pharmacological examinations of different drug prototype treatments. TSP1 is known to inhibit endothelium-dependent vasorelaxation via direct binding with endothelial CD47 receptor and co-binding with SIRPα protein; therefore, a CD47 blocking antibody was included in the assay as a standard to compare the efficiency of the proposed CD47-based non-immunogenic drug candidate and SIRPα blocking antibody to neutralize TSP1-mediated impairment of vasorelaxation. A prophylactic protocol design was represented, in which the vessel rings were incubated with CD47 blocking antibody, or different doses of the proposed CD47-based drug candidate or SIRPα antibody for a short pre-treatment period of 15 min, followed by co-incubation with a pathological level of TSP1 (4.4 nM of Pierce^TM^—TSP1) for another 1 h, was applied to investigate whether these pharmacological approaches were able to protect aortic vessels from TSP1 modulation. The CD47-based drug prototype substantially blocked the modulating effect by TSP1, as demonstrated through significant improvement in vasorelaxation in the presence of TSP1, while showing no significant difference from the cohort that was pre-treated with CD47 blocking antibody; meanwhile, SIRPα antibody failed in preserving appreciable vasorelaxation after TSP1 addition.

As TSP1 was demonstrated to impair endothelium-dependent vasorelaxation in as short as 15 min [5], a modified therapeutic protocol was developed to study the pharmacological effectiveness of the anti-TSP1 drug candidates in experimental conditions simulating pre-existing pathological levels of TSP1. Restoration of vasorelaxation in this post-treatment scenario following acute TSP1 challenge (applied as brief exposure to TSP1 for 15 min) was investigated, at various dose levels of drug candidates. Only the CD47-based drug candidate demonstrated its potential as a therapy, under ex vivo simulated pathological conditions, effectively reversing TSP1-diminished vasorelaxation. The representative bioassay data support the validity of this ex vivo model to evaluate the pharmacological vasoactivity profile. This would determine if proposed drug candidate(s) can potentially serve as an effective TSP1-antagonist(s), in order to qualify for further pre-clinical investigation, using the corresponding diseases animal model required for each specific indication. The method described here not only efficiently identified a new TSP1-specific drug prototype, but also helped to determine the effective dose range of the CD47-based drug candidate [13].

Adapted from the standard ex vivo myograph protocols, our protocol presents several practical benefits, especially regarding TSP1-related applications. The pre-treatment protocol in the presence of TSP1 only, utilizing TSP1 as the primary vasodilation antagonist, would be considered a more cost-effective and efficient approach/alternative for pharmacological baseline screening of vasodilators and/or vasoconstrictors, compared with utilizing expensive and complex transgenic animal models of TSP1-related pathologies. Owing to multiple TSP1-related vascular interactions with endothelial and smooth muscle cells, this pre-treatment protocol—utilizing healthy murine vessels—would also be very useful to pharmacologically evaluate several drug molecules and proteins that influence TSP1-modulation of vascular effects on native/normal tissue, to be compared later under pathological conditions. In addition to the relatively standard pre-treatment scenario, we have adopted a post-TSP1 treatment set-up of this ex vivo myograph-based protocol. In this new variation, an acute challenge by TSP1 for 15 min is introduced, followed by treatment with the candidate drug for 45 min. This post-treatment scenario has been useful in therapeutically screening anti-TSP1 drug candidates at various dose levels [13].

The flexible and facile pharmacological protocol presented here can be modified to any other type of tubular structure suitable for multiwire myograph receiving different soluble treatments and/or under diverse conditions [10,11,29]. For example, human pulmonary arteries from subjects with or without end-stage pulmonary hypertension were evaluated for the baseline endothelium-dependent and -independent responses to vasorelaxants; moreover, a similar pre-treatment protocol with antibody was applied to examine baseline vasorelaxation affected by tissue-resident TSP1 [10]. Automated multiwire myograph systems have been repeatedly used to measure the reactivity of isolated airways, specifically the trachea [30,31]. It should be noted that the optimal resting tension of the vessel to be tested is affected by a variety of factors, including subject species, age, diet, and genetic background of the experimental animals. In order to obtain the maximal active response, the optimal resting tension needs to be pre-determined, following a described process by others [32], or using the DMT normalization module included in the myograph system along with LabChart Pro Modules of DMT Normalization [33].

A notable limitation of the current protocol is the viability time frame for mounted vessels, particularly concerning their endothelial responsiveness. Different from in vitro assays, which generally can be paused at certain step(s) and stored for later processing, this ex vivo myograph-based protocol must be completed all in one experimental session, right after initial set-up. It is recommended that test vessels be freshly excised, which is quite important when endothelium-based proteins/functions are being investigated. With only a single layer of delicate endothelial cells, this experimental protocol should not continue to use test vessels, if mounted for longer than 6 h overall. Our laboratory observed somewhat reduced responsiveness to acetylcholine-induced endothelium-dependent vasorelaxation of isolated vessel (such as mouse thoracic aorta) when mounted for longer than 6 h on myograph. Observing this time window would merit superior consistency in response data. In general, prepared vessels can remain viable for about 6–9 h after mounting into the myograph, which is applicable for studying endothelium-independent vasoreactivity, owing to the relative robustness of multiple vascular smooth muscle cell layers.

## Figures and Tables

**Table 1:** Concentrations and volumes of acetylcholine working stocks applied for endothelium-dependent vasorelaxation assay.

Acetylcholine, Working Stock Concentration (M)	Volume of Working Stock to Be Added (µL)	Acetylcholine, Final Concentration (M)
1 × 10^−6^	5	1 × 10^−9^
1 × 10^−5^	1.0	3 × 10^−9^
1 × 10^−5^	3.5	1 × 10^−8^
1 × 10^−4^	1.0	3 × 10^−8^
1 × 10^−4^	3.5	1 × 10^−7^
1 × 10^−3^	1.0	3 × 10^−7^
1 × 10^−3^	3.5	1 × 10^−6^
1 × 10^−2^	1.0	3 × 10^−6^
1 × 10^−2^	3.5	1 × 10^−5^

## Data Availability

The data presented in this study are available on request from the corresponding authors.
